# Photoacoustic Imaging for Image-Guided Gastric Tube Placement: Ex Vivo Characterization

**DOI:** 10.3390/s25051597

**Published:** 2025-03-05

**Authors:** Samuel John, Yeidi Yuja Vaquiz, Nikhila Nyayapathi, Loay Kabbani, Anoop Nilam, Jonathan F. Lovell, Nicole A. Wilson, Yan Yan, Mohammad Mehrmohammadi

**Affiliations:** 1 Imaging Physics, The University of Texas MD Anderson Cancer Center, Houston, TX 77030, USA; sjohn11@mdanderson.org; 2 Department of Biomedical Engineering, Hajim School of Engineering and Applied Sciences, University of Rochester, Rochester, NY 14627, USA; yyujavaq@u.rochester.edu (Y.Y.V.); yan_yan1@urmc.rochester.edu (Y.Y.); 3 Department of Imaging Sciences, School of Medicine and Dentistry, University of Rochester Medical Center, Rochester, NY 14642, USA; nnyayapa@ur.rochester.edu; 4 Vascular Surgery, Henry Ford Health System, Detroit, MI 48202, USA; lkabban1@hfhs.org; 5 Department of Biomedical Engineering, School of Engineering and Applied Sciences, University at Buffalo, Buffalo, NY 14260, USA; anoopnil@buffalo.edu (A.N.); jflovell@buffalo.edu (J.F.L.); 6 Departments of Surgery, Pediatrics, & Biomedical Engineering, School of Medicine and Dentistry, University of Rochester Medical Center, Rochester, NY 14642, USA; nicole_wilson@urmc.rochester.edu

**Keywords:** gastrostomy tube, ultrasound, photoacoustic, image-guided, fiber, needle, contrast agent, optical properties

## Abstract

Over 250,000 gastrostomy tubes (G-tubes) are placed annually in the United States. Percutaneous endoscopic gastrostomy (PEG) is the most widely used clinical method for placing G-tubes within the stomach. However, endoscope detectability is limited due to the scattering of light by tissues. Poor organ visibility and low sensitivity of the palpation techniques cause blind needle insertions, which cause colon/liver perforations, abdominal bleeding, and gastric resections. Additionally, imaging artifacts and the poor distinguishability between water-filled tissues make ultrasound (US) imaging-based techniques incompatible with G-tube placement. The risk of ionizing radiation exposure and the confinement of fluoroscopy to radiology suites limits its bedside utility in patients. Considering these limitations, we propose to design a safe, point-of-care integrated US and photoacoustic (PA) imaging system for accurate G-tube placement procedures, for a broad spectrum of patients, and to characterize the system’s effectiveness. Our proposed technology utilizes a clinically safe contrast agent and a dual-wavelength approach for precise procedures. Our ex vivo tissue studies indicated that PA imaging accurately differentiates the different organs at specific wavelengths. Our characterization studies revealed that PA imaging could detect lower concentrations of Indocyanine Green (ICG) dye coating the colon wall, minimizing the risk of ICG dye-related toxicity and providing safer G-tube placements.

## 1. Introduction

Gastrostomy tube (G-tube) placement is a critical procedure for pediatric patients unable to maintain adequate oral intake due to different conditions, such as congenital anomalies, neuromuscular disorders, failure to thrive, and chronic illnesses. Each year, thousands of children require G-tubes to support their nutritional needs, and to enable growth and development while improving their quality of life [1]. Pediatric G-tube placements differ significantly from the adult procedures due to anatomical and physiological differences, and to heightened concerns over minimizing invasive techniques and radiation exposure [2]. Current methods for pediatric G-tube placement include percutaneous endoscopic gastrostomy (PEG) [3], laparoscopic-assisted techniques, and open-surgical approaches. PEG [4] and laparoscopic methods are preferred over fluoroscopy-guided placements to avoid the children’s exposure to ionizing radiation [5,6]. However, PEG procedures face other challenges, such as a reduced precision in identifying the optimal puncture site due to the scattering of endoscopic light through the abdominal wall and difficulty differentiating the stomach from other adjacent organs [3,7]. These limitations can lead to complications such as bleeding and may cause the perforation of other surrounding organs. Moreover, while providing better visualization, laparoscopic-assisted techniques require additional incisions and specialized surgical expertise, increasing procedural complexity and risks such as infections and longer recovery times [8,9].

**Figure 1:**
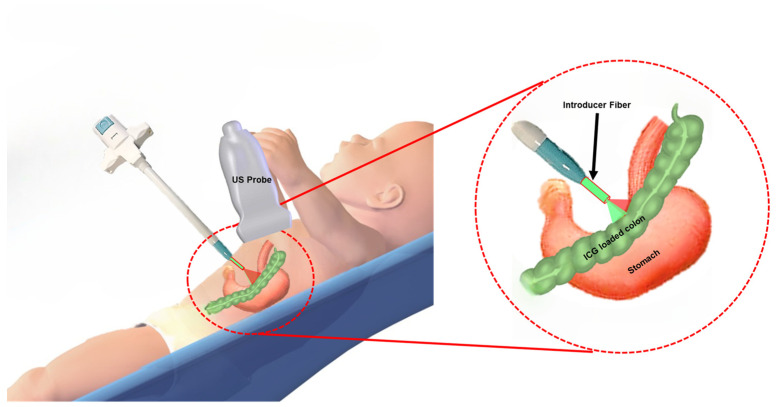
Illustration of the integrated US and PA-guided G-tube placement system. The relative variation in the PA signal intensity, due to the absorption properties of the exogenous and endogenous contrast agents, allows us to detect and differentiate between the stomach and the colon.

To address these challenges, there is a critical need for a minimally invasive, radiation-free imaging system to guide the G-tube placement in pediatric patients with greater accuracy and safety. This system should effectively differentiate tissue types in the needle’s trajectory and provide real-time guidance to avoid organ damage. Recent advances in ultrasound (US) and photoacoustic (PA) imaging technologies offer promising solutions. Studies of the literature have revealed that PA imaging has been used as a navigational tool in various medical applications. PA imaging uses a combination of non-ionizing laser light excitation and ultrasound emission to probe the optical properties of tissues. Integrating the functional PA information with US-probed structural tissue information aims to develop a non-invasive, easy-to-adapt, point-of-care platform for image-guided procedures. This study aims to develop a novel integrated US and PA-guided gastrostomy tube (G-tube) placement system for delivering G-tubes in pediatric patients in a safe and precise manner. Also, the study focuses on characterizing the system’s effectiveness, based on the exogenous contrast agent detection sensitivity as a function of distance and Indocyanine Green (ICG) dye concentration. By combining the high-resolution capabilities of the US with the tissue-specific contrast of PA imaging [10,11,12], the system can identify the puncture needle’s location and differentiate between the stomach and surrounding organs, such as the transverse colon, in real-time (Figure 1) [13,14]. Incorporating a food-grade, biocompatible contrast agent further enhances tissue distinction, thereby addressing the limitations of PA imaging in environments with similar optical properties. Unlike traditional approaches, this method eliminates the need for additional incisions or radiation-based imaging, offering a less invasive and safer option for pediatric patients. The proposed system leverages compact and portable components, including a diode laser and a clinical US transducer, enabling bedside deployment. This innovation can reduce procedural complexity, improve safety, and minimize hospitalization for pediatric G-tube placements, ultimately enhancing patient outcomes and addressing a critical, unmet clinical need.

## 2. Materials and Methods

### 2.1. PA Imaging for Evaluating Different Tissue Compositions

When the introducer fiber carrying the pulsed laser light of a given wavelength (λ) is placed close to an organ, a PA amplitude P(λ) is generated at the introducer tip:

(1) $$P\left(\lambda \right)=\Gamma *{µ}_{a}\left(\lambda \right)*F$$

where Γ corresponds to the Grüneisen parameter, describing the thermoelastic efficiency of the medium and µ_a_ refers to the absorption coefficient of the tissue being imaged. The laser fluence is denoted by F. P(λ_1_) is generated at wavelength λ_1_= 800 nm, selected to match the peak optical absorption (µ_a1_) of the clinically safe contrast agent (ICG) dye coating the colon wall. Similarly, a PA amplitude P(λ_2_) is generated at the introducer tip when the laser is pulsed at the wavelength (λ_2_= 532 nm), matching the absorption coefficient of the blood chromophores present in the stomach wall. Research studies have indicated that the stomach wall has double the absorption compared to the colon when illuminated at a pulsed laser wavelength of 532 nm [15,16].

Given the internal light illumination in our setup, the laser fluence can be easily computed as the laser light exiting the fiber illuminates a small region at the target tissue. The external illumination-based approach faces challenges in determining the laser fluence due to the light diffusion through heterogeneous tissue layers. Given that the pulsed laser-coupled introducer fiber illuminates a small region in front of it, the illuminated area can be easily computed, and the fluence can be determined. Hence, the fluence was kept constant at both wavelengths. The PA signal amplitude can be directly related to the absorption coefficient of the tissue chromophore when tuned to the chromophore-specific wavelength. Therefore, the ratio between the two generated PA amplitudes can differentiate the different organs at the specified wavelengths. Colon wall detectability is defined as:

(2) $$\frac{P\left(\lambda_{1}\right)}{P\left(\lambda_{2}\right)}=\frac{{µ}_{a1}\left(\lambda_{1}\right)}{{µ}_{a2}\left(\lambda_{2}\right)}>1$$

Stomach wall detectability is defined as:

(3) $$\frac{P\left(\lambda_{1}\right)}{P\left(\lambda_{2}\right)}=\frac{{µ}_{a1}\left(\lambda_{1}\right)}{{µ}_{a2}\left(\lambda_{2}\right)}<1$$

### 2.2. Integrated US and PA Image-Guided G-Tube Placement System

**Figure 2:**
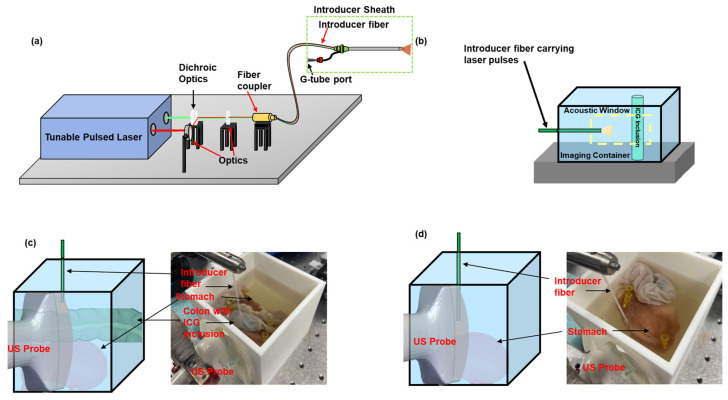
Schematic of the (**a**) integrated US and PA image-guided G-tube placement system. (**b**) Schematic of the experimental setup to characterize the minimum concentration of ICG needed to generate a detectable PA signal and characterize the intensity of the PA signal as a function of distance. Validating the organ detection capability of PA imaging to detect the (**c**) colon and (**d**) the stomach, in an ex vivo animal model.

The prototype of the proposed integrated system was designed and developed by combining the pulsed laser beam from a tunable pulsed laser system (PhocusCore, Opotek, Carlsbad, CA, USA) (pulse energy = 200 μJ, λ = 532 nm, 800 nm and repetition rate = 10 Hz) with an introducer fiber (Ø =1000 μm NeverTouch, Angiodynamics) through dichroic optics (Ø1”, DMSP567) (Figure 2a). A pulsed laser wavelength of λ = 532 nm is used because it matches the absorption properties of the blood vessels of the stomach. Similarly, a pulsed laser wavelength of λ = 800 nm was used based on the absorption properties of the exogenous contrast agent (ICG). A high-frequency linear array US transducer (L11-4V, 128 elements, Verasonics, Kirkland, WA, USA) and the programmable digital US research platform (Vantage 128, Verasonics, Kirkland, WA, USA) provided the co-registered US and PA images.

### 2.3. Characterization of the Sensitivity of PA Imaging to Detect ICG

Several research groups have utilized ICG as a contrast agent for assessing the blood flow to the bowel walls [17], identifying anastomotic perfusion [18] and anastomotic leakage [19]. ICG has also been used as a contrast agent to highlight the blood vessels in brain angiography applications [20] and tumor identification [21] in cancer procedures. The sensitivity of PA imaging to detect the ICG dye was characterized by imaging different ICG concentrations at a fixed distance from the introducer fiber, and imaging a fixed concentration of ICG at varying distances from the fiber. The concentration sensitivity of PA imaging was evaluated by imaging ICG (IO1250, Pfaltz & Bauer, Waterbury, CT, USA) concentrations of 0.5, 0.25, 0.125, 0.0675, and 0.03375 mg/mL in a water solvent, within an US-transparent tube (ID: 1 mm, Vacutainer Blood Collection Set, BD Vacutainer, Franklin Lake, NJ, USA), at a fixed distance of 9 mm from the introducer fiber tip (Figure 2b). The distance sensitivity of PA imaging was evaluated by imaging US-transparent tube-filled ICG at a specific concentration of 0.25 mg/mL in a water solvent, at distances of 1, 2, 3, 4, 6, 8, and 12 mm from the introducer fiber tip (Figure 2b). Both studies were performed in a water bath with 0.2% cellulose added to generate a turbid medium. Based on preliminary UV-VIS spectroscopy data, they were imaged at a wavelength of λ = 780 nm. US and PA signals were acquired by positioning the US transducer coupled to the acoustic window. The PA signal in each set of experiments was normalized to its series.

### 2.4. Evaluating the Organ Detection Capability of PA Imaging in an Excised Rabbit Tissue

The organ detection capability of PA imaging was further evaluated in an ex vivo animal model by imaging tissue samples, including an adult rabbit’s colon measuring 100 mm in length, and a stomach, whose cross-section measured approximately 7 cm^2^. The colon was thoroughly flushed with a 1:10 diluted PBS solution (10X PBS, Bio-Rad, Hercules, CA, USA) to remove fecal matter completely. A cylindrical-shaped, ICG-inclusion-based gelatin phantom, measuring 110 mm in diameter, was inserted into the colon to simulate the ICG dye coating of the colon wall. The 10% wt/wt gelatin inclusion (G6144, Sigma Aldrich, St. Louis, MO, USA) had a 0.25 mg/mL concentration of ICG (IO1250, Pfaltz & Bauer, Waterbury, CT, USA). The tissue samples were positioned in a 100 × 100 mm plastic container, with a US gel-coated optical window, accommodating the US transducer probe (L11-4V). The container was filled with the diluted PBS solution to mimic the scattering properties of tissues and to serve as a medium for ultrasound imaging. The container held the samples over an 8% wt/wt gelatin block (G6144, Sigma Aldrich, St. Louis, MO, USA). Colon detectability was evaluated by imaging the colon, positioned on top of the stomach, with wavelengths (λ = 532 nm, 780 nm, pulse energy = 450 μJ, repetition rate = 10 Hz) (Figure 2c). The introducer fiber was positioned perpendicular to the excised tissues, at the particular distances of 3.3 mm and 5.5 mm. Stomach detectability was evaluated by imaging the stomach tissue at the above wavelengths, at 1.7, 4.5, and 9 mm distances from the introducer fiber (Figure 2d).

## 3. Results

### 3.1. Sensitivity of PA Imaging to Detect ICG

**Figure 3:**
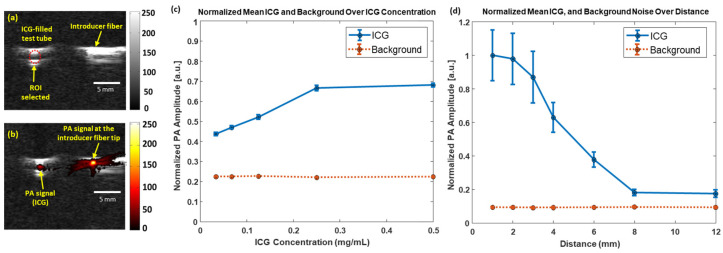
The high sensitivity of PA imaging used to detect different ICG concentrations at varying distances. (**a**) The US image highlighting the ROI, where (**b**) the PA signal is generated from different ICG concentrations. (**c**) The detection of ICG increases at 4 mm from the fiber tip, (**d**) while the signal plateaus (or quenches) at concentrations higher than 0.25 mg/mL. Both scenarios were imaged at a wavelength λ = 780 nm. The solid blue line indicates the variation in the PA signal as a function of (**a**) concentration and (**b**) distance. The dotted orange line indicates the background noise.

The ability of PA imaging to detect the minimum concentration of ICG at proximal distances to the colon was evaluated by analyzing the PA signal amplitude at the selected ROI (Figure 3a,b) when advanced toward different concentrations of the ICG sample. Figure 3c illustrates that the PA signal increases linearly with ICG concentration, plateauing at 0.25 mg/mL. This indicates that higher concentrations, such as 0.5 mg/mL, are unnecessary for effective PA detection. Also, a detectable PA signal, observed at the lowest ICG concentration of 0.03375 mg/mL, further validates the dependence of the PA signal on the absorption coefficient of its interacting medium. Figure 3d revealed the PA signal variation as a distance function. The PA signal decreases when the distance between the introducer fiber and the target medium increases. A strong, detectable PA signal is observed at distances of less than 6 mm, and is four times larger than the background, which refers to the system’s noise floor. Hence, PA imaging can be a non-invasive tool to avoid organ perforations.

### 3.2. Evaluating the Dual-Wavelength PA Approach in an Ex Vivo Animal Model

**Figure 4:**
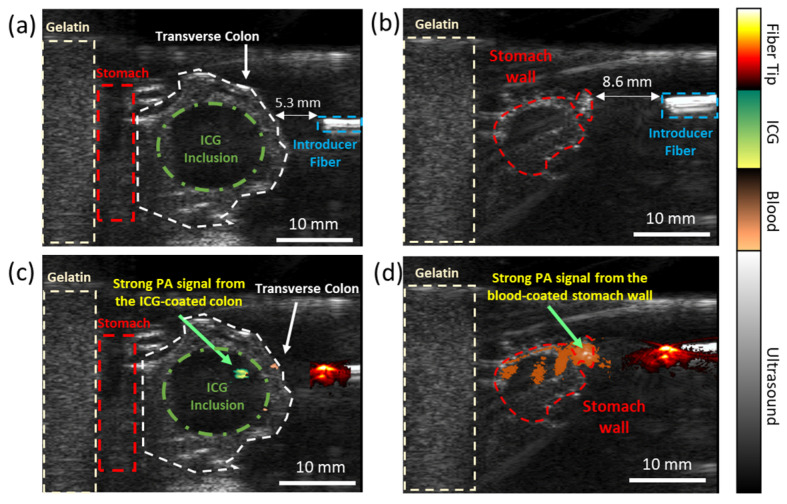
Dual-wavelength PA imaging approach for detecting different types of organs in an ex vivo animal model: the US image indicating the presence of (**a**) a colon and (**b**) no colon positioned between the stomach and the introducer fiber tip. Co-registered USPA images indicate the presence of the colon and the stomach tissue through color coding: (**c**) green color indicates the presence of an ICG dye-loaded colon and (**d**) orange color indicates the presence of a vascularized stomach wall. The ‘hot’ colormap indicates the PA signal generated at the introducer fiber tip. The different PA signal intensities at specific wavelengths are a non-invasive indicator for avoiding organ perforations.

The dual-wavelength approach was further validated using an excised rabbit’s transverse colon and stomach tissues in an ex vivo setting. The food-grade ICG dye-loaded colon and the stomach were illuminated using two wavelengths, particularly λ = 780 nm, corresponding to the absorption peak of ICG, and λ = 532 nm, corresponding to the absorption peak of blood. The introducer fiber was positioned perpendicular to the tissues, in a specific arrangement mimicking the different scenarios occurring during G-tube placement when a colon (Figure 4a) or no colon (Figure 4b) is in the path of the stomach. The ratio of the PA signal at different wavelengths acts as a differentiating indicator for organ detectability. Figure 4c reveals a strong PA signal at the colon tissue when the introducer fiber is positioned at least 5 mm away from it. Also, a strong PA signal is observed at the stomach tissue when the introducer fiber is positioned <8 mm away from it (Figure 4d). Moreover, different types of tissues can be identified along the introducer fiber’s trajectory at a resolution of (0.2 × 0.2 mm), providing real-time guidance to avoid colonic injuries.

## 4. Discussion

The proposed system offers a unique and safe approach to G-tube placement procedures. In contrast to the scattering of endoscopic light through tissue layers, the internal illumination mode-enabled PA imaging technique transduces laser light pulses into acoustic waves, which experience minimal acoustic attenuation from the tissue layers. The developed system does not suffer from varying fluence because the pulsed laser light does not pass through the tissue layers, and only illuminates a small region from the introducer fiber tip. Our previous characterization studies have shown that the variation in the PA signal is independent of fiber orientation and experiences minimal attenuation from tissues at relevant imaging depths [22]. High-contrast PA images of the fiber tip will improve instrument visualization and reduce tracking-related errors. Organ differentiation was performed by tuning the pulsed laser wavelength of the introducer fiber to the absorption coefficient of the external contrast agent (ICG) coating the colon wall. In clinical cases, the air-filled colon limits the visibility of ultrasound imaging, causing complications. However, the intravenously delivered ICG dye coats the vascularized colon wall, enabling the developed PA system to detect the ICG dye-coated air-filled colon [23]. The low ICG concentration needed to generate a detectable PA signal minimizes the risk of complications caused by high ICG dye concentrations [24]. The radiopaque contrast agent, Barium, delivered via enema, used in fluoroscopic procedures to highlight the colon, induces longer hospital durations, and serves a niche patient population [25]. Hence, our proof-of-concept studies utilize a clinically approved ICG dye to highlight the colon. However, as an alternative to the ICG dye, research has been conducted to develop low-cost, food-grade, and bio-compatible contrast agents for PA imaging [26,27,28]. These contrast agents, such as food-grade activated charcoal [28] and ingestible roasted barley [27], have been used for visualizing and mapping the intestines in studies on mice [27,29]. They do not have a distinct peak absorption like ICG dye, but their absorption at near-infrared and 1064 nm excitation is significantly larger than that of blood [27,28]. Moreover, the penetration depth of PA imaging at 1064 nm is desirable due to low optical tissue scattering [30]. Also, the high contrast PA signal from the target from background tissue (often blood) allows for organ detectability [31]. The acquisition of PA signals through a clinically used US transducer and minimal modifications in the imaging sequence to display co-registered USPA images promote the easy translation of this technology into a clinical environment. Easy integration of optical fibers to existing clinical devices, such as introducer needles and balloon catheters, enables smooth adaptability to clinicians, as the proposed technology does not entirely change the percutaneous approach. Accurate fiber tip tracking at significant imaging depths allows the proposed technology to cater to a broad patient audience (including high BMI patients).

## 5. Conclusions

The integrated US–PA image-guided G-tube placement system offers a novel and non-invasive approach that can potentially lower the complexity and complications associated with G-tube placement. The development of this G-tube placement system has implications for easy integration with standard US-based imaging systems, patient safety, and hospital cost savings. High-contrast PA images of the fiber tip will allow the surgeon to safely navigate intraoperatively. The risk of organ puncture associated with standard PEG procedures can be reduced substantially, as PA imaging allows the user to detect the organs through the dual-wavelength approach. PA imaging also eliminates the risk associated with ionizing radiation and strong magnets, typical of other G-tube placement techniques. Using clinically approved, safe contrast agents for highlighting the organs favors easy adaptability and caters to a broad patient range. Developing a point-of-care system will eliminate the risk of cross-contamination among patients, offer a more cost-effective approach, and can be used in critical care units. Future studies will involve the characterization and validation of food-grade contrast agents for organ detectability in animal models and cadaver studies.

## Data Availability

The data supporting the findings of this study are available upon request from the authors.
